# Association between Polymorphism *rs1799732* of *DRD2* Dopamine Receptor Gene and Personality Traits among MMA Athletes

**DOI:** 10.3390/genes12081217

**Published:** 2021-08-06

**Authors:** Marta Niewczas, Anna Grzywacz, Katarzyna Leźnicka, Krzysztof Chmielowiec, Jolanta Chmielowiec, Agnieszka Maciejewska-Skrendo, Pavel Ruzbarsky, Jolanta Masiak, Wojciech Czarny, Paweł Cięszczyk

**Affiliations:** 1 Faculty of Physical Education, University of Rzeszów, 3 Towarnickiego St., 35-959 Rzeszów, Poland; mniewczas@ur.edu.pl (M.N.); wojciechczarny@wp.pl (W.C.); 2 Independent Laboratory of Health Promotion, Pomeranian Medical University in Szczecin, 11 Chlapowskiego St., 70-204 Szczecin, Poland; 3 Faculty of Physical Education, Gdansk University of Physical Education and Sport, 1 K. Górskiego St., 80-336 Gdansk, Poland; katarzyna.leznicka@awf.gda.pl (K.L.); cieszczyk@poczta.onet.pl (P.C.); 4 Department of Hygiene and Epidemiology, Collegium Medicum, University of Zielona Góra, 28 Zyty St., 65-046 Zielona Góra, Poland; chmiele@vp.pl (K.C.); chmiele1@o2.pl (J.C.); 5 Institute of Physical Culture Sciences, University of Szczecin, 40B Piastów St., 71-065 Szczecin, Poland; maciejewska.us@wp.pl; 6 Faculty of Sports, University of Presov, 15, 17. novembra St., 080 01 Prešov, Slovakia; pavel.ruzbarsky@unipo.sk; 7 Neurophysiological Independent Unit, Department of Psychiatry, Medical University of Lublin, 1 Aleje Racławickie St., 20-059 Lublin, Poland; jolantamasiak@wp.pl

**Keywords:** mixed martial arts (MMA), dopamine, D2 receptor, personality traits, genetics

## Abstract

Four factors—namely, harm avoidance, novelty seeking, reward addiction and persistence—represent the nature of temperament that is not genetically determined in itself. It was shown in earlier studies that a strong propensity to look for novelty or a tendency to engage in risky behavior is correlated with genetic variants in the area of the genes encoding dopamine receptors. Therefore, the aim of this study is to determine whether there is a relationship between personality traits and genetic variants in the area of the *DRD2* dopamine receptor gene in MMA athletes. The participants consisted of 85 mixed martial arts (MMA) athletes and 284 healthy, non-MMA male participants. Their personality traits were measured using the Revised Temperament and Character Inventory. Blood was collected for genetic assays and all samples were genotyped using the real-time PCR method. We observed a statistically significant effect of a complex factor of the *DRD2 rs1799732* genotype on MMA participants’ control and reward dependence. Engaging in high-risk sport may be associated with several personality characteristics. The *DRD2 rs1799732* polymorphism may be associated with reduced harm avoidance in martial arts athletes, thereby modulating athletes’ predisposition to participate in high-risk sport.

## 1. Introduction

Being a professional athlete is very demanding. An increased level of competition is always connected with the occurrence of more detailed factors determining success achievement. Elite athletes very often have to act under enormous pressure, which is generally connected with different psychological features influencing the achievement of success at the top level, in comparison with others that are less successful [1].

High resistance of the nervous system and unique personality features such as: persistence, motivation to act, will power and conscientiousness allow athletes to prepare effective strategies for dealing with physical and psychological loads, anxiety or pain [2]. The role of particular personality features reveals itself in the strongest form when a person acts in stressful conditions and carries out tasks requiring special mobilization of the body. 

Studies of innate psychological features among combat athletes suggest lower sensitivity and higher resistance of the central nervous system when compared with non-athletes [3]. Competitors who are less sensorily sensitive deal better with tension, lack of comfort, time pressure and physical discomfort. The control of emotional agitation contributes to emotion control and energy management to obtain the maximal results, especially in situations of great stress during sport competition. Athletes who practice individual sports are more introverted in comparison with athletes in team sports [4]. The anxiety of the competitor is connected with a continuous increase in the level of physical fitness and maintenance of effective muscle tension, but also coordination difficulties and disturbances in concentration, especially when an athlete observes they lack control in a specific situation.

Meta-analysis describing the problem of personality variety in different groups of athletes and non-athletes indicates a higher level of extraversion and lower level of neuroticism among athletes in comparison with the general population [5]. Most research shows that the individual effectiveness of competitors is positively correlated with extraversion and negatively with neuroticism [6,7,8].

Previous reports regarding the temperamental characteristics of athletes, using the Temperament and Character Inventory (TCI) by Cloninger, have observed significant connections between impulsiveness, impetuousness and novelty seeking (NS) [9,10]. Han et al. in the conducted study observed the predictive role of persistence in terms of the possibility of winning [11]. Cloninger, in his report (1986), observed that individuals with higher persistence are more predisposed to continuous task performance without immediate successive reward or boosting [12].

Studies conducted by Hollis et al. [13] emphasized how individual personality traits could modulate sport injury susceptibility. Authors have noted that rugby players with increased concussion risk achieve higher impulsiveness scores. Both impulsiveness and aggression were related to concussion history in that same group of athletes [14].

It is probable that relations between the genetically conditioned temperament and environmentally conditioned character highly modulate the will to participate in and continue with sport training, as well as modifying the tolerance to strenuous effort, which as a result, may affect the achievement of success in this area [15].

Four factors—namely, harm avoidance (HA), novelty seeking (NS), reward dependence (RD) and persistence—represent the character of one’s temperament, which is not genetically conditioned by itself, but is moderately heritable and time stable [16]. Personality traits connected with a strong will for novelty seeking (NS) or a tendency to undertake risky behaviors were previously correlated with genetic variants in the area of the genes coding dopamine receptors. Dopamine is one of the main neurotransmitters within the behavioral approach system [17] involved in regulating the brain systems that control the cognitive and emotional decision processes that underlie both extraversion and neuroticism [18]. Subtypes of dopamine receptors D2 and D4 participate in dopamine neurotransmission and may modulate memory, behavior and executive functions. 

Research confirms that variation in the genes regulating the dopaminergic brain system modulate differences in processing in the striatum, prefrontal cortex areas and limbic system, which are centrally involved in decision-making, reinforcement learning and risk assessment [19,20].

Thus, the aim of this paper is to determine whether there are connections among temperament, personality traits and genetic variants in the area of the dopamine receptor gene *DRD2* in MMA athletes.

## 2. Materials and Methods

### 2.1. Research Groups 

Both the sport and control groups in this study were of Caucasian origin and lived in a single Polish region. The experiment was based on a group of 85 healthy (no prior history of substance dependency or psychosis) Polish mixed martial arts (MMA) athletes aged 25.95 ± 6.69. The study population was classified as ‘sub-elite’ (participants in international competitions with no less than eight years of training experience). Various methods were used to obtain the samples, including targeting national teams and providing information to national coaching personnel and athletes attending training camps. Controls included 284 healthy (non-dependent and non-psychosis), non-MMA-athletic Polish male volunteers aged 22.89 ± 4.77. All athletes and controls were Caucasian to reduce the possibility of racial gene skewing and to overcome any potential problems due to population stratification (Table 1).

### 2.2. DNA Isolation and Genotyping

A standard procedure of collecting venous blood was applied to obtain genomic DNA that was used for genotyping in accordance with the real-time PCR method. Genotyping of rs1799732 in the *DRD2* gene was performed by fluorescence resonance energy transfer in a LightCycler 480 II System (Roche Diagnostic, Basel, Switzerland) according to the standard manufacturer’s protocols.

### 2.3. Psychometric Tests

The following psychometric tests were also performed. The Revised Temperament and Character Inventory (TCI-R) is a self-report questionnaire assessing the four aspects of temperament (harm avoidance, novelty seeking, reward dependence and persistence) and three higher-order character dimensions (self-directedness, cooperativeness and self-transcendence). Temperament refers to individual differences in perception-based habits and skills that are regulated by the limbic system and measured by four independently inherited dimensions that are moderately stable throughout life: novelty seeking (NS) refers to a tendency toward exploratory activities in response to novelty and is hypothesized to be mediated by a dopaminergic behavioral activation system; harm avoidance (HA) refers to pessimistic worrying in anticipation of problems and is hypothesized to be mediated by a serotonergic behavioral inhibition system; reward dependence (RD) is defined as a tendency to maintain behaviors in response to reward by others and is mediated by a noradrenergic behavioral maintenance subsystem; persistence(PS) is an independent dimension and refers to a tendency to perseverance despite frustration and fatigue [12,16].

### 2.4. Statistical Analysis

The *DRD2 rs1799732* genotype’s distribution was tested according to the Hardy-Weinberg equilibrium (HWE) with the following HWE software: https://wpcalc.com/en/equilibrium-hardy-weinberg/ (accessed on 3 June 2021).

The analyzed variables did not have a normal distribution, so a Mann-Whitney U test was applied to determine the differences in the analyzed traits of novelty seeking, harm avoidance, reward dependence, self-management, cooperation ability and self-transcendence skills.

Not all assumptions required for analysis of variance (ANOVA) analysis were met. The assumption of a normal distribution was not fulfilled for all dependent variables, but the variance was the same (Levene’s test *p* > 0.05). Because the numbers of subjects in the groups were large, a multivariate analysis 2 × 3 factorial ANOVA was decided upon. The test was used to reveal associations of novelty seeking, harm avoidance, reward dependence, self-management, cooperation ability and self-transcendence skills in the MMA and control groups with the *DRD2 rs1799732* polymorphism (control × personality traits; MMA subjects × genetic feature).

The frequencies of the genotypes and alleles of the *DRD2 rs1799732* polymorphism in the analyzed groups were compared by the chi-squared test. All analyses were performed using STATISTICA 13 (Tibco Software Inc., Palo Alto, CA, USA) for Windows (Microsoft Corporation, Redmond, WA, USA)

## 3. Results

The frequency distributions were in accordance with the HWE. There was no statistical differences between the MMA and control subjects (Table 2).

The *DRD2 rs1799732* genotypes’ and alleles’ frequencies in the studied sample did not differ between the subjects of the two analyzed groups (Table 3).

The means and standard deviations for novelty seeking, harm avoidance, reward dependence, self-management, cooperation ability and self-transcendence skills in the MMA and control subjects are presented in Table 3. In comparison with the controls, the case group subjects had significantly higher scores for self-management (M 26.59 vs. M 23.88, *p* < 0.0001) and lower scores for harm avoidance (M 9.92 vs. M 11.35, *p* = 0.019) and reward dependence (M 9.50 vs. M 10.47, *p =* 0.008; Table 4).

### 3.1. Reward Dependence and DRD2 rs1799732 

**Figure 1:**
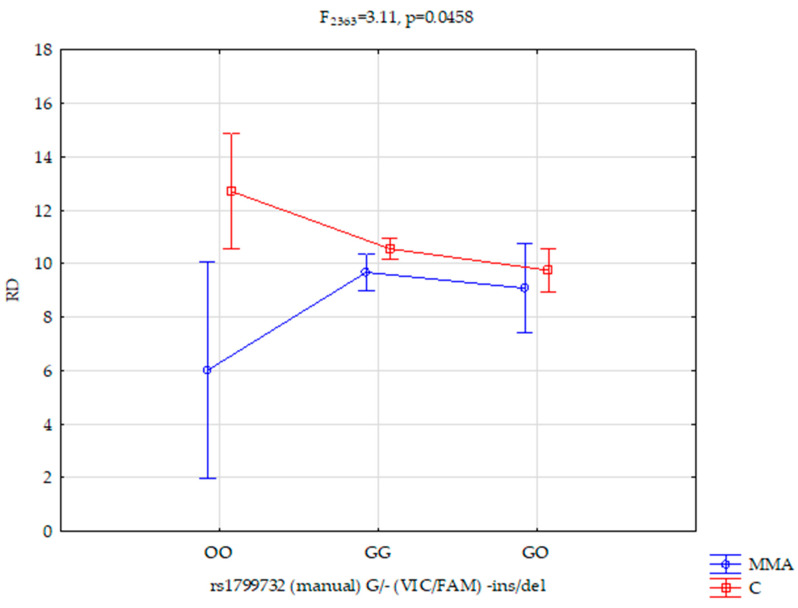
Interactions between *DRD2 rs1799732* and reward dependence (RD) for mixed martial arts (MMA) subjects and control groups.

The results of a 2 × 3 factorial ANOVA of the MMA and control subjects were found for reward dependence (F_1363_ = 10.53, *p* = 0.0013, η^2^ = 0.028; Table 5). The analysis had a 90% power to detect in the MMA and control subjects the effects of the studied reward dependence and the interaction (about 3% of the phenotype variance). We found a statistically significant effect of a complex factor of the *DRD2 rs1799732* genotype on reward dependence in both groups (F_2363_ = 3.11, *p* = 0.0458, η^2^ = 0.017; Table 5 and Figure 1). The analysis had a more than 59% power to detect the complex factor of MMA/control x *DRD2 rs1799732* and their interaction effect they exert (about 2% of the phenotype variance).

### 3.2. Cooperative Abilities and DRD2 rs1799732 

The results of a 2 × 3 factorial ANOVA of cooperative abilities were found for the MMA group in relation to the *DRD2 rs1799732* genotype (F_2363_ = 3.24, *p* = 0.0402, η^2^ = 0.018; Table 5). The analysis had a more than 61% power to detect the *DRD2 rs1799732* genotype’s effects in the studied group on their cooperative abilities, along with the interaction effect (about 2% of the phenotype variance).

## 4. Discussion

The goal of every athlete is to achieve the maximum level of body adaptation to exercise and significant sporting success. High-performance athletes are subject to enormous training loads, which can lead to an imbalance between training and regeneration, as well as having a negative impact on the athlete’s psyche.

Researchers generally agree that sporting development is based on mutual influences of athlete’s predispositions, environment, practice and training [21]. At the same time, when we consider sport talents, we can observe an increased tendency to limit the concentration on physiology and anthropometry and promote the psychological aspect as the key element of talent development [21,22,23]. However, there are only a few publications concerning the genetic conditioning of the psychological traits of athletes, despite great progress in the direction of understanding the genetics of physical fitness [24] and previous encouragement for psychological testing [25,26]. 

Motivational behavior is usually augmented in the event of increased reward-related activity and is connected with increased synaptic DA availability. Mutations in *DRD4* gene are connected with different behavioral phenotypes, for instance, risk-taking [27,28]. There are a few dozen polymorphic loci in a promoter region of *DRD4*. Research has, for instance, indicated that allele C of the *rs1800955* polymorphism is connected with novelty seeking and extraversion. The other polymorphism is a variation of tandem repeats (VNTR) of 48 base pairs in exon 3, ranging from 2-repeat (2R) to 11-repeat (11R) alleles [29]. People with a 7R allele engage in increased levels of physical activity [30,31,32] and are more sensitive to external factors [33,34,35]. The 7R allele is also connected with risky financial decisions [36,37], concentration deficit/hyperactivity [30,35], alcoholism [38] and over-sensitiveness [39]. Research on a group of MMA athletes showed that they have an increased frequency of 7R alleles in comparison with a control group [40].

In our research, we observed that the controls, in comparison with the case group subjects, scored significantly higher on the scale of self-management (M 26.59 vs. M 23.88, *p* < 0.0001), while at the same time, scoring lower on the scales of harm avoidance (M 9.92 vs. M 11.35, *p* = 0.019) and reward dependence (M 9.50 vs. M 10.47, *p* = 0.008; Table 4). What is more, we noted a statistically significant effect of a complex factor of the *DRD2 rs1799732* genotype on reward dependence in both groups (F_2363_ = 3.11, *p* = 0.0458, η^2^ = 0.020; Table 5 and Figure 1). It was found that the *DRD2 rs1799732* genotype was related to cooperation ability (F_2363_ = 3.24, *p* = 0.0402, η^2^ = 0.018; Table 5).

In this study, we observed a distinct connection between personality traits and genetic variants in the area of the dopamine receptor gene *DRD2* among MMA athletes. However, a similar study of 109 normal healthy people did not confirm the association of *DRD2* genotypes with TCI scales (NS, harm avoidance, reward dependence and PS) [41]. It is clear when we consider the aforementioned relation of the dopaminergic system with sport activity, but additionally, we have discovered new aspects of athletes’ characteristics and an importance of psychological features that not only predispose people to practice their chosen sport disciplines, but are also characteristic of successful athletes. In a study by Abrahams et al. (2019) among professional rugby players, the *DRD4 rs1800955 CC* genotype and the inferred *DRD2* (*rs12364283–rs1076560*)–*DRD4* (*rs1800955*) A-C-C allele combination were over-represented in the control group. The results indicated that the *rs1800955* C allele was related to reduced concussion susceptibility. The authors hypothesized that the C allele may stimulate *DRD4* expression, increasing the D4 receptor’s availability to dopamine. Thus, the C allele acts as a neuro-protective response against concussion injury by inhibiting ‘risk-taking’ behavior [42].

There are several forms of evidence that the development of elite athletes is genetically conditioned. However, our present progress in understanding the molecular bases of cognitive skills and personality features among athletes is still at its very beginning. Further research could open up the area to more possibilities of sports psychogenetics applications with the usage of new DNA technologies (for example, whole genome sequencing, genome-wide association study (GWAS), epigenetic, transcriptomic and proteomic profiling) and bioinformatics, to analyze the influence of genes on athletes’ behaviors.

## 5. Conclusions

Engaging in a high-risk sport (martial arts) may be associated with several personality characteristics. In the present study, we found an interaction between the dopamine *DRD2 rs1799732* polymorphism and the athletic status in terms of harm avoidance and cooperation ability. The association with reduced harm avoidance in martial arts athletes, in particular, could modulate athletes’ predisposition to participate in high-risk sport. Behavioral studies of personality traits and their interactions with biological factors, such as personality genetics, constitute a new, promising area of research. Linking personality traits to the basics of inheritance could contribute to improved understanding of sports motivation and behavior.

## Figures and Tables

**Table 1:** Fundamental biological features of tested mixed martial arts (MMA) group and control group.

Feature	Mixed Martial Arts (MMA) *N* = 85	Control *N* = 284
Age	25.95 ± 6.69	22.89 ± 4.77
Body mass	83.07 ± 13.01	81.31 ± 10.76
Body height	180.09	181.76 ± 6.07
body mass index (BMI)	25.18	24.29 ± 3.92

**Table 2:** Hardy-Weinberg equilibrium of the *DRD2 rs1799732* in group of MMA subjects and controls.

Group	*DRD2 rs1799732*				
		Observed (Expected)	Alleles Frequency	χ^2^	*p* Value
MMA *N =* 85	GG	71 (69.8)	p allele freq (C) = 0.91 q allele freq (A) = 0.09	2.517	0.113
	GO	12 (14.5)			
	OO	2 (0.8)			
Controls *N* = 284	GG	229 (225.4)	p allele freq (C) = 0.89 q allele freq (A) = 0.11	4.870	0.027
	GO	48 (55.2)			
	OO	7 (3.4)			

*p*, statistical significance; χ^2^, chi-squared test result; *N*, number of subjects.

**Table 3:** Frequencies of genotypes and alleles of the *DRD2 rs1799732* polymorphism in MMA subjects and controls.

Group	*DRD2 rs1799732*				
	Genotypes			Alleles	
	GG N (%)	GO N (%)	OO N (%)	G N (%)	O N (%)
MMA *N* = 85	71 (0.84)	12 (0.14)	2 (0.02)	154 (0.91)	16 (0.09)
Controls *N* = 284	229 (0.81)	48 (0.17)	7 (0.02)	506 (0.89)	62 (0.11)
χ^2^ *p* value	0.383 0.826			0.313 0.576	

*p*, statistical significance; χ^2^, chi-squared test result; *N*, number of subjects.

**Table 4:** Analysis of novelty seeking, harm avoidance, reward dependence, self-management, ability to cooperate and self-transcendence in MMA subjects and controls.

	MMA	Control	UZ	*p* Value
	**(*N* = 85)** **M ± SD**	**(*N* = 284)** **M ± SD**		
Novelty Seeking	20.36 ± 5.44	20.27 ± 4.63	−0.152	0.879
Harm Avoidance	9.92 ± 5.00	11.35 ± 4.60	2.341	0.019 *
Reward dependence	9.50 ± 3.02	10.47 ± 2.91	2.641	0.008 *
Self-management	26.59 ± 3.92	23.88 ± 5.10	−4.347	0.00001 *
Cooperative abilities	19.87 ± 4.48	19,97 ± 4.63	0.224	0.8230
Self-transcendence skills	6.65 ± 3.61	7.06 ± 3.54	1.021	0.3069

M, mean; SD, standard deviation; UZ, U Mann-Whitney Z-test. * Statistically significant between-group differences.

**Table 5:** Results of 2 × 3 factorial ANOVA for MMA subjects and controls, incorporating novelty seeking, harm avoidance, reward dependence, self-management, ability to cooperate, self-transcendence skills and *DRD2 rs1799732*.

	*DRD2 rs1799732*					2 × 3-Factor ANOVA				
	MMA (*N* = 85) M ± SD	Control (*N* = 284) M ± SD	GG (*N* = 300) M ± SD	GO (*N* = 60) M ± SD	OO (*N* = 9) M ± SD	Full Model F (*p* Value)	Factor	F (*p* Value)	ɳ^2^	Power (Alpha = 0.05)
Novelty Seeking	20.36 ± 5.44	20.27 ± 4.64	20.28 ± 4.80	20.87 ± 4.87	16.88 ± 4.62	F_5363_ = 1.749 *p* = 0.1227 R^2^ = 0.023	intercept	F_1363_ = 714.78 (*p* < 0.0001 *)	0.663	1.000
							MMA/control	F_1363_ = 2.47 (*p* = 0.1162)	0.007	0.348
							*DRD2 rs1799732*	F_2363_ = 3.726 (*p* = 0.0250 *)	0.020	0.681
							MMA/control × *DRD2 rs1799732*	F_2363_ = 1.67 (*p* = 0.1900)	0.003	0.149
Harm Avoidance	9.92 ± 5.00	11.36 ± 4.60	11.07 ± 4.80	10.78 ± 4.53	11.33 ± 3.43	F_5363_ = 2.177 *p* = 0.0561 R^2^ = 0.029	intercept	F_1363_ = 274.07 (*p* < 0.0001 *)	0.430	1.000
							MMA/control	F_1363_ = 0.40 (*p* = 0.5265)	0.001	0.097
							*DRD2 rs1799732*	F_2363_ = 0.57 (*p* = 0.5606)	0.003	0.146
							MMA/control × *DRD2 rs1799732*	F_2363_ = 2.20 (*p* = 0.1121)	0.012	0.449
Reward Dependence	9.51 ± 3.02	10.47 ± 2.91	10.35 ± 2.86	9.62 ± 3.27	11.22 ± 3.63	F_5363_ = 3.581 *p* = 0.0036 * R^2^ = 0.047	intercept	F_1363_ = 515.08 (*p* < 0.0001 *)	0.586	1.000
							MMA/control	F_1363_ = 10.53 (*p* = 0.0013 *)	0.028	0.899
							*DRD2 rs1799732*	F_2363_ = 1.09 (*p* = 0.336)	0.006	0.242
							MMA/control × *DRD2 rs1799732*	F_2363_ = 3.11 (*p* = 0.0458 *)	0.017	0.597
Self-Management	26.59 ± 3.92	23.88 ± 5.10	24.39 ± 5.09	24.85 ± 4.57	26.00 ± 3.84	F_5363_ = 4.754 *p* = 0.0003 * R^2^ = 0.061	intercept	F_1363_ = 1280.54 (*p* < 0.0001 *)	0.779	1.000
							MMA/control ×	F_1363_ = 0.50 (*p* = 0.4810)	0.001	0.108
							*DRD2 rs1799732*	F_2363_ = 0.18 (*p* = 0.837)	0.001	0.077
							MMA/control × *DRD2 rs1799732*	F_2363_ = 0.95 (*p* = 0.387)	0.005	0.215
Cooperative Abilities	19.87 ± 4.48	19.98 ± 4.63	19.94 ± 4.62	19.45 ± 4.36	23.67 ± 3.94	F_5363_ = 2.461 *p* = 0.0328 * R^2^ = 0.0327	intercept	F_1363_ = 957.38 (*p* < 0.0001 *)	0.725	1.000
							MMA/control	F_1363_ = 1.82 (*p* = 0.1777)	0.005	0.270
							*DRD2 rs1799732*	F_2363_ = 3.24 (*p* = 0.0402 *)	0.018	0.616
							MMA/control × *DRD2 rs1799732*	F_2363_ = 2.75 (*p* = 0.0650)	0.015	0.542
Self-Transcendence Skills	6.66 ± 3.61	7.06 ± 3.54	7.01 ± 3.53	6.98 ± 3.78	5.67 ± 2.92	F_5363_ = 1.052 *p* = 0.3866 R^2^ = 0.014	intercept	F_1363_ = 147.62 (*p* < 0.0001 *)	0.289	1.000
							MMA/control	F_1363_ = 0.84 (*p* = 0.3583)	0.002	0.151
							*DRD2 rs1799732*	F_2363_ = 1.47 (*p* = 0.2304)	0.008	0.314
							MMA/control × *DRD2 rs1799732*	F_2363_ = 1.58 (*p* = 0.2071)	0.009	0.335

M, mean; SD, standard deviation. * Statistically significant between-group differences.

## Data Availability

Not applicable.
